# Malignant Extra Renal Rhabdoid Tumour Presenting as Central Airway Obstruction

**DOI:** 10.1155/2014/950869

**Published:** 2014-08-27

**Authors:** Karan Madan, Amanjit Bal, Ritesh Agarwal, Ashim Das

**Affiliations:** Departments of Histopathology and Pulmonary Medicine, Post Graduate Institute of Medical Education & Research (PGIMER), Sector 12, Chandigarh 160012, India

## Abstract

Rhabdoid tumours are one of the most aggressive childhood neoplasms associated with high mortality. The commonest age group affected is children less than five years of age. Rhabdoid tumour presenting as an endoluminal tracheal mass leading to central airway obstruction has not been previously reported. We describe the case of a 17-year-old male patient where malignant rhabdoid tumour masqueraded as bronchial asthma leading to a delayed diagnosis of upper airway obstruction by tracheal growth. Histopathological examination and immunohistochemistry confirmed the diagnosis of malignant rhabdoid tumour.

## 1. Introduction

Malignant rhabdoid tumour (MRT) is an uncommon and one of the most aggressive malignancies in infancy with reported mortality rate of 80 to 100% [1]. The usual site of occurrence is the kidney. MRTs were initially identified as a sarcomatous variant of the Wilms tumour but were subsequently demonstrated to have characteristic pathological features. In recent years, occurrence of rhabdoid tumours has been reported at a number of extra renal sites (extra renal malignant rhabdoid tumour) [2]. According to the best of our knowledge, this is the first reported case of malignant rhabdoid tumour presenting as tracheal growth and central airway obstruction. The present case also highlights the fact that upper airway obstruction by tumour can masquerade as and be mistreated as bronchial asthma. Therefore whenever, on clinical examination findings, a fixed/monophonic wheeze or stridor is audible, upper airway obstruction should be suspected and investigated using appropriate radiological/bronchoscopic examinations.

## 2. Case History

### 2.1. Clinical Findings

A 17-year-old male patient presented to the emergency room with history of progressively increasing shortness of breath over five-month duration. Shortness of breath had markedly worsened over two days prior to presentation. There was associated history of wheezing and the patient had been managed as a case of bronchial asthma over the course of the illness using inhaled bronchodilators without any improvement in symptoms. There was no history of fever, expectoration, hemoptysis, or foreign body inhalation. The patient had lost weight and appetite had reduced over the same period. On examination, the patient appeared anxious, pulse rate was 120/min, blood pressure was 130/90 mm Hg (pulsus paradoxus was present—40 mm Hg), and respiratory rate was 24/min with visible use of accessory muscles of respiration. On examination, a prominent inspiratory stridor was audible. The rest of the physical examination was unremarkable. Chest radiograph was essentially normal. Arterial blood gas analysis demonstrated respiratory acidosis (Ph 7.34, PO_2_ 55.9 mm Hg, PaCO_2_ 59.1 mm Hg, HCO_3_^−^ 32 meq/L, and SpO_2_ 87% while breathing room air). Patient was administered high flow oxygen using a venturi mask with which the SpO_2_ improved to 93%.

**Figure 1:**
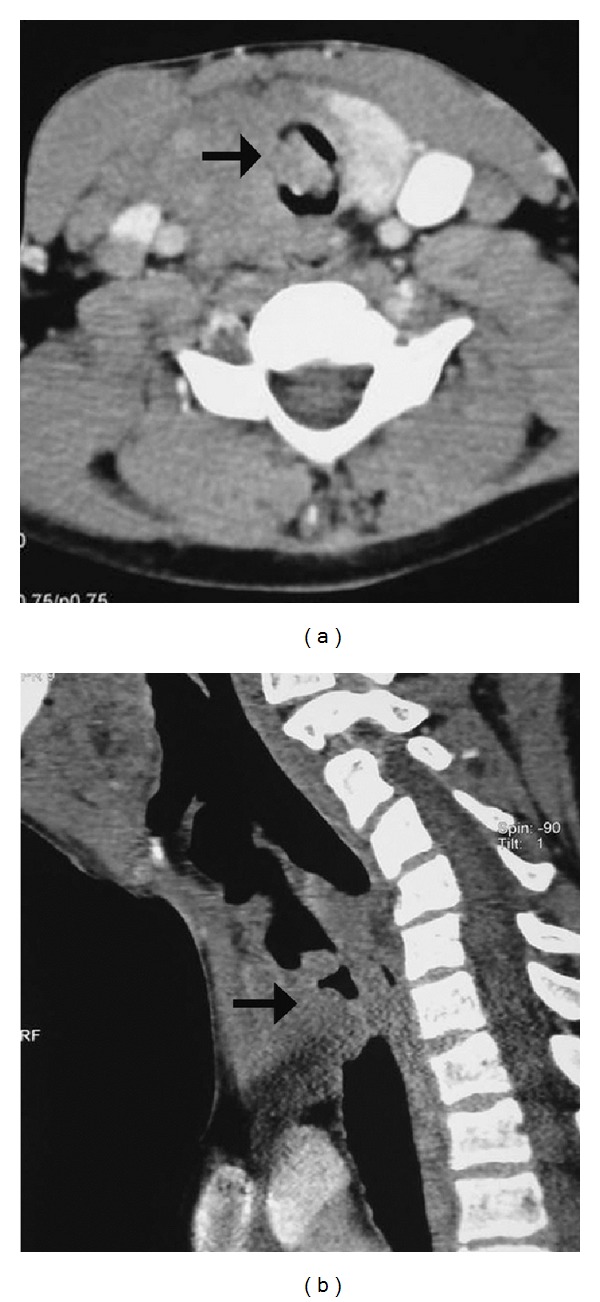
CT scan image of the neck (axial section (a)) demonstrating an endoluminal soft tissue mass in the upper tracheal lumen arising from the right tracheal wall which is causing near complete occlusion of the upper trachea (sagittal section (b)).

In view of a clinical possibility of upper airway obstruction, urgent CT examination of the thorax was performed. CT showed an endoluminal mass in the upper trachea with extraluminal component arising from the right anterolateral wall of the upper trachea causing nearly 80% narrowing of the tracheal lumen (Figures 1(a) and 1(b)). Patient was planned for an urgent rigid bronchoscopic debulking of the tracheal tumor. During the procedure, a large fleshy tumor was visible in the immediate subglottic region causing near complete tracheal occlusion. Attempt was made to core out the tumor using the barrel of the rigid bronchoscope. Before the tumor could be completely removed, the patient sustained cardiac arrest and resuscitation was unsuccessful. The tracheal growth tissue obtained at the time of rigid bronchoscopy was submitted for histopathological examination.

### 2.2. Pathology Findings

**Figure 2:**
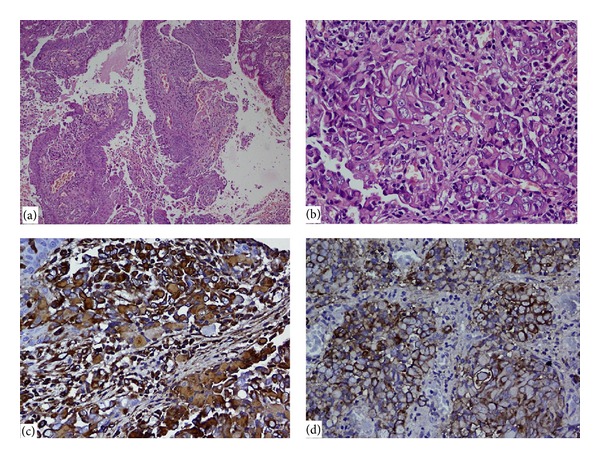
Pathology examination: (a) haematoxylin and eosin stained sections revealed respiratory lining epithelium, with subepithelium showing a tumour. (b) The tumour cells were predominantly arranged in sheets and were large, round to oval, having abundant pale eosinophilic to clear cytoplasm, eccentric large nucleus, and prominent nucleoli. ((c) and (d)) On immunohistochemistry, the tumour cells were positive for epithelial membrane antigen (EMA) and vimentin, respectively.

Haematoxylin and eosin stained sections revealed respiratory lining epithelium, with subepithelium showing a tumour (Figure 2(a)). The tumour cells were predominantly arranged in small clusters and sheets and around blood vessels. The tumour cells were large, round to oval, having abundant pale eosinophilic to clear cytoplasm, eccentric large nucleus, and prominent nucleoli (Figure 2(b)). Cytoplasm showed eosinophilic globular inclusions in perinuclear location. On immunohistochemistry, the tumour cells were positive for epithelial membrane antigen (EMA), cytokeratin, and vimentin (Figures 2(c) and 2(d)). Tumour cells were negative for leukocyte common antigen (LCA), S-100, WT1, desmin, myogenin, neuron specific enolase (NSE), CD34, CK7, and TTF-1 excluding lymphoma, melanoma, Wilms' tumor, rhabdomyosarcoma, neuroblastoma, epithelioid sarcoma, and rhabdoid large cell carcinoma of lung, respectively. Final diagnosis of the malignant extra renal rhabdoid tumour was given.

## 3. Discussion

Originally described in the kidney and reported as “rhabdomyosarcomatous variant of Wilms tumor,” the rhabdoid tumour is now defined as a distinct clinicopathologic entity with a characteristic clinical course and histopathologic, immunohistochemical, and cytogenetic profile. The incidence of ERRT was reported as 0.15 per million children <15 years [1]. The central nervous system is the most frequent site of ERRT; however, it is reported in various sites of body [2]. Extra renal rhabdoid tumors have been described in the liver, brain, tongue, neck, chest, heart, pelvis, extremities, and several other sites. Three reports have highlighted rhabdoid differentiation in the lung tumours; however, it has not been described as endoluminal growth in the trachea [3–5].

This patient presented with upper airway obstruction masquerading as bronchial asthma for which he was treated. The treatment options for central airway tumors include surgery and radiotherapy, and in those with inoperable or emergent airway obstruction, therapeutic rigid bronchoscopy is the treatment of choice [6]. In this patient, the rigid bronchoscopy was done to relieve the obstruction, and the tumour tissue which was removed revealed extra renal rhabdoid tumour on pathology examination. The histologic hallmark of ERRT is the presence of “rhabdoid” cells: large polygonal cells with perinuclear, globular, eosinophilic cytoplasmic inclusions and vesicular nuclei that contain a single prominent nucleolus. Architecturally, it dominantly shows solid pattern but trabecular and pseudoalveolar patterns may also be seen.

The histogenesis of MRT is uncertain, and on immunohistochemistry, it shows common mesenchymal and epithelial differentiation [7]. Vimentin is the most consistently expressed marker. Epithelial membrane antigen (EMA) and cytokeratin are the next most frequently expressed markers. The cell derives its name from the resemblance to rhabdomyoblasts, although current evidence does not support a myogenic origin as these tumours lack immunoreactivity for muscle differentiation with MyoD1 and myogenin.

Due to its potential to occur at different sites, it can create a diagnostic challenge. The pathological differential diagnosis includes epithelioid sarcoma, melanoma, and rhabdomyosarcomas exhibiting rhabdoid-type inclusions. Also rhabdoid morphology has been reported in different tumours, and it is difficult to determine whether they represent pure extra renal MRT or represent rhabdoid morphology only in focal areas of a tumour with recognizable phenotype. Carcinomas of various types have rhabdoid features of which renal cell carcinoma is the most common. In this case the rhabdoid variant of large cell carcinoma of the lung, needed exclusion. Large cell carcinomas with rhabdoid morphology show expression of epithelial markers and vimentin like MRT, however in addition they also express CK7 and thyroid transcription factor-1 (TTF-1) [8]. Cells with rhabdoid features may be seen in epithelioid sarcoma, especially the “proximal type” epithelioid sarcoma. Both tumours show morphological and immunohistochemical overlap with coexpression of epithelial markers and vimentin; however epithelioid sarcomas express CD34, a marker which is typically absent in MRT [9]. Malignant melanomas can also show rhabdoid histology and lack of pigment making its distinction from MRT difficult. However, malignant melanomas express S-100, HMB45, and Melan A which is lacking in MRTs.

Recently with the advent of molecular techniques, the defining feature of ERRTs is an aberration (mutation or deletion) of the INI1 gene located at chromosome 22q11.1 [10]. The accurate diagnosis of ERRT is important for both prognostication and correct treatment. This is especially relevant with the increasing availability of targeted therapies.
